# Long-Term Outcome After Retro-Areolar Versus Peri-Tumoral Injection of Superparamagnetic Iron Oxide Nanoparticles (SPIO) for Sentinel Lymph Node Detection in Breast Cancer Surgery

**DOI:** 10.1245/s10434-019-07239-5

**Published:** 2019-03-04

**Authors:** Fredrik Wärnberg, Evelina Stigberg, Christine Obondo, Helena Olofsson, Shahin Abdsaleh, Madeleine Wärnberg, Andreas Karakatsanis

**Affiliations:** 1 Department of Surgical Sciences, Uppsala University, Uppsala, Sweden; 2 Department of Surgery, University Hospital Wishaw, Wishaw, UK; 3 Department of Immunology, Genetics and Pathology, Uppsala University, Uppsala, Sweden; 4 Department of Clinical Pathology, Uppsala University Hospital, Uppsala, Sweden; 5 Aleris, Mammography Unit, Uppsala, Sweden; 6 Department of Surgery, Uppsala University Hospital, Uppsala, Sweden

## Abstract

**Background/Objective:**

SPIO is effective in sentinel node (SN) detection. No nuclear medicine department is needed, and no allergic reactions have occurred. This study aimed to compare retro-areolar and peri-tumoral SPIO injections regarding skin staining, detection rates and number of SNs.

**Methods:**

Data on staining size, intensity and cosmetic outcome (0–5; 0 = no problem) were collected by telephone interviews with 258 women undergoing breast conservation. SN detection and the number of SNs were prospectively registered in 332 women.

**Results:**

After retro-areolar and peri-tumoral injections, 67.3% and 37.8% (*p* < 0.001) developed skin staining, with remaining staining in 46.2 vs. 9.4% after 36 months (*p* < 0.001). Initial mean size was 16.3 vs. 6.8 cm (*p* < 0.001) and after 36 months, 6.6 vs. 1.8 cm^2^ (*p* < 0.001). At 75.1% of 738 interviews, staining was reported paler. After retro-areolar injections, cosmetic outcome scored worse for 2 years. The mean (median) scores were 1.3(0) vs. 0.5(0) points, and 0.2(0) vs. 0.1(0) points, at 12 and 36 months, respectively. Overall detection rates were 98.3% and 97.4% (*p* = 0.43) and the number of SNs 1.35 vs. 1.57 (*p* = 0.02) after retro-areolar and peri-tumoral injections. Injection, regardless of type, 1–27 days before surgery increased detection rates with SPIO, 98.0% vs. 94.2% (*p* = 0.06) ,and SN numbers, 1.56 vs. 1.27 (*p* = 0.003).

**Conclusion:**

SPIO is effective and facilitates planning for surgery. Peri-tumoral injection reduced staining with a similar detection rate. Staining was not considered a cosmetic problem among most women. Injecting SPIO 1–27 days before surgery increased the detection rate by 3.8% and increased the number of SNs by 0.3.

## Background

Superparamagnetic iron oxide nanoparticles (SPIO) have comparable detection rates as the dual technique using Technetium^99^ (Tc^99^) and Blue Dye (BD) for sentinel node (SN) detection.^1^,^2^ SPIO has not been associated with allergic reactions and eliminates the need for nuclear medicine facilities, simplifying operative planning. The short half-life of Tc^99^ and the risk for allergic reactions related to BD mandate that tracers are administered peri- or intraoperatively. However, SPIO may be injected up to 4 weeks before a SNB. At Uppsala University Hospital, SPIO has been used as the sole SN detection method for 2 years in most cases.^3^

As with BD, SPIO injection may cause skin staining for more than a year. This is seen almost exclusively after breast-conserving surgery (BCS). For BD, 41% of patients have been reported with staining after 12 months and up to 8.6% after 36 months.^4^,^5^ To avoid skin staining, the injection technique was modified, and instead of injecting SPIO behind the areola, it was injected deeper, close to the tumour.^1^

The purpose of the study was to compare the rate of skin staining after retro-areolar and peri-tumoral injections and how the different injection techniques related to patient-experienced cosmetic outcome. The detection rates and the number of removed SNs also were studied.

## Patients and Methods

**Fig. 1:**
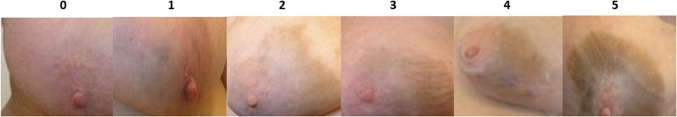
Self-reported intensity of staining after a SPIO injection was based a scale from zero to five. The women received this intensity scale per mail or per letter

Women in whom SPIO was used, from April 2014 to November 2017, were included. Those undergoing BCS were analysed regarding SN detection rates and skin staining, whereas those with mastectomies were analysed regarding detection rates only. The retro-areolar injection of SPIO (2 ml Sienna+^®^ in 3 ml of NaCl) was given at least 20 min before surgery followed by a 5-min massage. Later, when SPIO was injected up to 4 weeks before surgery, massage was optional. During the latter part of the study period, SPIO was injected close to palpable tumours or in the peritumoral area for nonpalpable tumours. If the transcutaneous magnetic signal in the axilla was low, BD was added according to the surgeon’s decision. If no SN was found, the decision to perform an axillary clearance, an axillary biopsy, or no staging was left to the surgeon. Age, body mass index (BMI), type of surgery, tumour size and grade, number of SNs, and lymph node status were documented. The size of skin staining was recorded at the first postoperative visit, 3 weeks after surgery. Women with a skin stain were thereafter telephone interviewed every third month. The size of the staining, intensity, and cosmetic outcome was self-assessed. At first, only change of intensity was described, but later women classified the intensity of the staining according to a Likert item scale from 0 to 5, based on photos of selected cases mailed to the women (Fig. 1). In the absence of a relevant, validated questionnaire, women were asked to evaluate the cosmetic outcome of the staining on a Likert item scale from 0 to 5 (0 = not a problem, 1 = slight problem, 2 = minor problem, 3 = clearly a problem, 4 = considerable problem, 5 = important problem). The self-assessment gave us the subjective views of the women. Follow-up was ended when the staining was gone. The study was approved by the ethics committee at Uppsala University; Dnr:2014/073 with amendments 2014/073/01 and 2014/073/02. The manuscript was prepared according to the STROBE statement.^7^

### Statistical Analysis

Staining was analysed only in women with BCS, but analyses of SN detection were done in all women, BCS, and mastectomies. Comparisons between cohorts were conducted using parametric tests, when appropriate. Data measured on a Likert scale were analysed using nonparametric procedures. Univariate analyses of correlation were performed; variables with significance or trend to significance (*p* < 0.1) were tested in a multivariate regression model. SPSS^®^ version 23.0 (IBM, Armonk, NY) was used for statistical analyses.

## Results

In total, 337 women were included, undergoing 340 operations. All women who were injected with a retro-areolar injection of SPIO between April 2014 and October 2016 constituted our first cohort. All of these women were part of earlier studies conducted at our hospital.^1^,^3^,^6^ Women injected with a peritumoral injection between November 2016 and November 2017 were included in our second cohort. Some of these women were part of the Monos study.^3^

Breast-conserving surgery was performed in 257 women (1 bilateral BCS). Six women had conversion to mastectomy, and staining was only registered at the first postoperative visit. Seven women (all in the peritumoral cohort) did not have a SN biopsy (SNB), even though SPIO was injected, because they were part of the SentiNot trial.^20^ Those seven women were only included in the skin-staining analysis. Eighty women had a primary mastectomy (2 bilateral). In the retro-areolar cohort, there were 110 BCS and 67 mastectomies. In the peritumoral cohort, there were 147 BCS and 15 mastectomies. Cohorts are presented in Table 1. In the retro-areolar cohort, tumours were significantly larger and of higher grade. However, difference in size was not significant when looking at BCS only (16.1 and 16.3 mm, *p* = 0.86).

**Table 1:** Patient and tumor characteristics in breast cancer patients undergoing sentinel lymph node biopsy using superparamagnetic iron oxide nanoparticles (SPIO) for sentinel node detection

Patient and tumor characteristics	SPIO injection site				*p* value	
	Retro-areolar, *n* = 177		Peritumoral, *n* = 163			
	Mastectomy + BCS SN detection	BCS skin staining	Mastectomy + BCS SN detection	BCS skin staining		
Number of surgical operations	177	110	156	148	Mastectomy + BCS	BCS
Age, years (mean, range)	63.7 (32–89)	63.1 (34–82)	63.0 (34–82)	62.8 (39–79)	0.543^a^	0.820^a^
BMI, kg/m^2^ (mean, range)	27.6 (17.4–42.1)	25.8 (17.4–42.1)	26.7 (18.0–41.6)	26.0 (18.0–41.6)	0.795^a^	0.778^a^
Tumour size, mm (mean, range)	22.7 (2–123)	16.1 (2–80)	18.2 (2–103)	16.3 (2–63)	0.006^a^	0.864^a^
Nuclear grade						
1	26 (14.7%)	18 (16.4%)	44 (28.2%)	43 (29.1%)	0.026^b^	0.008^b^
2	86 (48.6%)	59 (53.6%)	61 (39.1%)	59 (39.9%)		
3	50 (28.2%)	24 (21.8%)	44 (28.2%)	42 (28.4%)		
Missing	15 (8.5%)	9 (8.2%)	7 (4.5%)	4 (2.7%)		
Histopathology						
Ductal invasive	124 (70.1%)	79 (71.8%)	124 (79.5%)	113 (76.4%)	0.647^b^	0.859^b^
Lobular invasive	25 (14.1%)	6 (14.5%)	20 (12.8%)	8 (12.2%)		
DCIS/LCIS	17 (9.6%)	9 (8.2%)6 (5.5%)	6 (3.8%)	11 (7.4%)		
Other	11 (6.2%)		6 (3.8%)	6 (4.1%)		

In the two cohorts, a retro-areolar injection or a deeper peritumoral injection were used, respectively. All patients (breast-conserving surgery or mastectomy) and those with breast-conserving surgery only are presented in separate columns

*BCS* breast-conserving surgery

^a^Student’s *t* test

^b^Chi square test

### Skin Staining

**Fig. 2:**
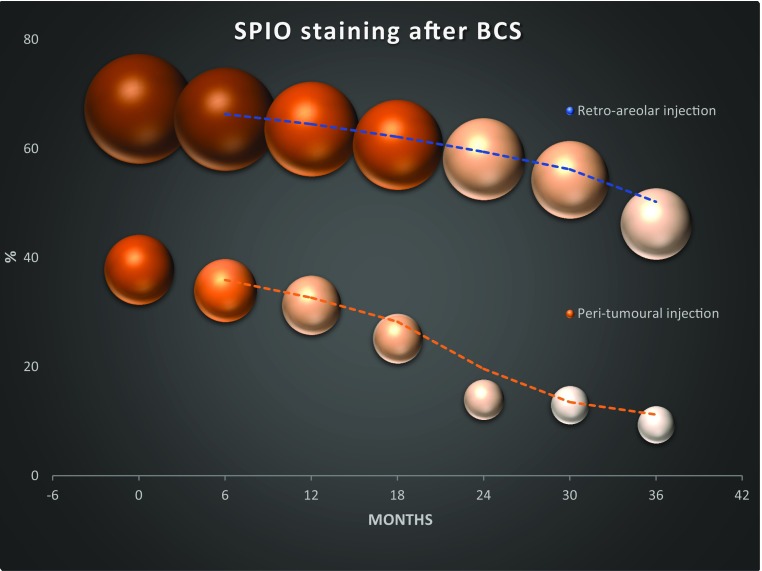
Incidence and self-assessed size of skin staining after a retro-areolar or peritumoral injection of superparamagnetic iron oxide nanoparticles (SPIO) for sentinel lymph node detection in women with breast-conserving surgery

After BCS, 74 of 110 (67.3%) had a skin staining after a retro-areolar and 56 of 148 (37.8%) after a peritumoral injection (*p* < 0.001). Including all women, the mean size of staining was 16.3 (range 2-100) cm^2^ and 6.8 (range 1–100) cm^2^ after retro-areolar and peritumoral injections (*p* < 0.001), at the first visit. Including only those 130 with an actual staining, the mean size was 24.2 and 17.9 cm^2^ (*p* = 0.02), respectively. After 6, 12, 24, and 36 months, 65.4%, 63.6%, 58.1%, and 46.2% had a remaining staining after retro-areolar injections and 34.0%, 31.3%, 14.0%, and 9.4% after peritumoral (*p* < 0.001 at 36 months). Size diminished successively over time (Fig. 2; Table 2). In a univariate analysis, including injection type and time, age, BMI, and tumour size, only retro-areolar injection and age were statistically significantly related to staining (data not shown). Both age and injection type were significantly related to skin staining in a multivariate analysis, including these factors: age, relative risk (RR) 1.04 [95% confidence interval (CI), 1.01–1.06], and retro-areolar injection, RR 3.58 (2.10–6.08). Intensity was reported by the women to be paler at 554 of 738 interviews (75.1%). After introducing the intensity-scale, 46 women of 75 with a remaining stain answered, and 15 of those answered twice, with a 3-month interval. The mean score of intensity, regardless of injection type, was 2.8, 1.7, and 0.9 points at 6–12, 13–24, and 25–36 months, respectively. In those with two successive scorings, the reported intensity score was 1.2 points less at the second scoring. No difference in intensity of the staining was found at 36 months after retro-areolar or peritumoral injections (*p* = 0.60).

**Table 2:** Incidence and self-assessed size of skin staining after a retro-areolar or peritumoral injection of superparamagnetic iron oxide nanoparticles (SPIO) for sentinel lymph node detection in women with breast-conserving surgery (BCS)

	3 weeks	6 months	12 months	24 months	36 months
*Retro*-*areolar injection*					
All women undergoing BCS	110	107	107	107	104
Skin staining (%)	67.3	65.4	63.6	58.1	46.2
Size cm^2^ (mean)	16.3	13.8	11.6	8.7	6.6
Cosmetic outcome, 0–5 (mean)	–	1.3	1.3	0.6	0.2
Only those women with a remaining stain at each time point (number)	74	70	68	61	48
Size cm^2^, (mean)	24.2	21.1	18.2	15.1	14.0
Cosmetic outcome, 0–5 (mean)	–	2.0	2.0	1.0	0.4
*Peritumoral injection*					
All women undergoing BCS	148	147	147	121	117
Skin staining (%)	37.8	34.0	31.3	14.0	9.4
Size cm^2^ (mean)	6.8	5.1	4.5	2.1	1.8
Cosmetic outcome, 0–5 (mean)	–	0.5	0.5	0.2	0.1
Only those women with a remaining stain at each time point (number)	56	50	46	17	11
Size cm^2^ (mean)	17.9	15.1	14.3	15.1	18.8
Cosmetic outcome, 0–5 (mean)	–	1.4	1.5	1.5	1.0

Cosmetic outcome was self-assessed according to a Likert item scale 0–5: 0 = not a problem; 1 = slight problem; 2 = minor problem; 3 = clearly a problem; 4 = considerable problem; 5 = important problem

Self-assessed cosmetic outcome (0-5 points) was worse after retro-areolar compared with peritumoral injections at 12 and 24 months: mean (median) 1.3 (0) vs. 0.5 (0) points (*p* < 0.001) and 0.6 (0) vs. 0.2 (0) points (*p* = 0.02). However, the difference was gone after 36 months: 0.2 (0) vs. 0.1 (0) for retro-areolar and peritumoral injections, respectively (*p* = 0.49). Analysing women with an actual stain at each time point showed no statistically significant differences between the two injection types (data not shown). Women with a higher BMI scored lower at all time points, regardless of injection type, but the differences were not statistically significant (data not shown). Younger women (< 60 years) scored worse than older women (≥ 60 years) during the first 2 years, but the difference was gone at year 3. Mean (median) for < 60 years were 2.4 (3), 2.4 (3), 1.3 (1), and 0.2 (0) points and for ≥ 60 years 1.4 (1), 1.4 (1), 0.7 (0), and 0.4 (0) points at 6, 12, 24, and 36 months, respectively (*p* < 0.001, *p* = 0.04, *p* = 0.06, and *p* = 0.84). There was no statistically significant difference in self-assessed cosmetic outcome comparing 27 women with small and less intensive staining or large and more intensive staining after 12 months (0–2 points and < 15 cm^2^ vs. 3–5 points and ≥ 15 cm^2^; *p* = 0.55).

### Sentinel Lymph Node Biopsy

There were 333 operations with a goal for a SNB. Four of those had had earlier axillary surgery and were excluded from analyses. The overall SN detection rate was 97.9% (322/329). In four women, SNs were detected by BD only. All of these had a retro-areolar injection of SPIO on the day of surgery. Five women had palpable macrometastatic lymph nodes when entering the axilla, despite a negative axillary ultrasound, and intraoperative frozen section confirmed metastases. Detection rates for those with retro-areolar and peritumoral injections were 98.3% and 97.4% (*p* = 0.43), respectively. Excluding those with palpable metastasis and just looking at SN detection by SPIO showed no significantly different detection rates for retro-areolar and peritumoral injections: 95.9% (164/171) versus 97.4% (149/153) (*p* = 0.50). Nonpalpable SN metastasis were all detected by SPIO, regardless of injection type or timing: 32/32 for retro-areolar and 19/19 for peritumoral injections.

The SN detection rate with SPIO was 98.0% (199/203) after injection 1-27 days before surgery and 94.2% (113/120) after an injection on the day of surgery (*p* = 0.06); 1–7 days before surgery 97.5%, 8–14 days 100% and 15–27 days 97.5%. No difference in detection rates was found between injection types, comparing women who had their injection at similar timepoints before surgery.

The mean number of removed SNs were 1.35 and 1.57 for those with retro-areolar and peritumoral injections, respectively (*p* = 0.02). In those injected at the day of surgery, the number of SNs were 1.21 after retro-areolar and 1.53 after peritumoral injections (*p* = 0.07). The mean number of SNs was significantly higher in those injected 1 to 27 days before surgery regardless of injection type: 1.56 and 1.27, respectively (*p* = 0.003). The mean number of SNs after injections at days 1–7, 8–14, and 15–27 before surgery was 1.74, 1.53, and 1.52, respectively.

Women with a lower BMI (< 25 kg/m^2^) had higher SN detection rate with a similar number of SNs, regardless of injection type: 100% versus 93.3% (*p* < 0.02), and 1.45 versus 1.46, respectively (*p* = 0.31). Women with mastectomy or BCS had similar SN detection rates: 98.7% versus 97.5% (*p* = 0.53). The mean number of SNs removed was 1.29 at mastectomy and 1.51 at BCS (*p* = 0.04), but numbers were similar if SPIO was injected at similar time points (day 0; 1.20 vs. 1.17, *p* = 0.29; day 1-27; 1.40 vs. 1.60, *p* = 0.28).

## Discussion

Sentinel node identification using SPIO is comparable to TC^99^ and BD.^1^,^2^ In this study, SPIO was injected in two different ways and at different time points. A peritumoral compared to a retro-areolar injection, resulted in less and smaller skin staining with comparable detection rates. Injecting the SPIO before the day of surgery increased SN detection rate by approximately 4% (*p* = 0.06) with 0.3 more removed SNs. Most women did not consider skin staining to be a cosmetic problem.

More than 300 women were followed for up to 3 years. All data were registered prospectively, and the cosmetic outcome of skin staining was patient-assessed. Patient reported outcomes have been shown to be more sensitive in reflecting patient satisfaction than outcomes reported by clinicians.^8^ Even if the cosmetic and intensity scales have not been validated, data clearly showed that staining successively got paler, and most women did not consider the staining a major problem. The use of Likert items, which is the most recommended scale for patient-related outcome measures allowed for a report in a comprehensive manner for the women.^9^ Staining may persist, but at 3 years it was regarded as “no” or “minor cosmetic problem” by 88% of the women. Mean and median scorings by all those with a stain were 0.5 and 0. Today the intensity scale (Fig. 1) can be shown for patients with an explanation that after 3 years the intensity was scored as 0.9, if the stain remained.

A peritumoral injection reduced the incidence of staining, and the cosmetic outcome was initially better compared with retro-areolar injections. The staining disappeared earlier after a peritumoral injection, and after 12 months the mean score was 0.5 (median 0). The BD also results in staining of the skin, but little is reported about patient-experienced outcome. Govaert et al. noticed that none of 33 women followed for 18 months reported the blue staining a cosmetic or psychological problem.^5^ In the study by Gumus et al., none of 115 women with remaining blue staining after 12 months reported a cosmetic or psychological problem.^4^

In concordance with what has been reported for the dual technique, different injection sites resulted in similar SN detection rates.^10^ Interestingly, injecting the SPIO 1-27 days before surgery enhanced detection rates from 94.2 to 98.0%. However, this was only of borderline statistical significance. This agrees with experimental data that demonstrated that SPIO concentration in SN related to time from injection.^11^ The detection rate when the SPIO injection was made on the day of surgery was however better than reported detections rates for BD alone.^12^ The number of SNs increased from 1.21 to 1.53 when injecting earlier, but still, this is fewer removed SNs than in most reported studies.^13^,^14^ The possibility to inject the tracer up to 4 weeks before surgery is novel. It provides flexibility and makes logistics easier. Thus, SPIO may be injected at an ordinary outpatient visit when surgery is planned, and there is no need for an extra visit to the nuclear medicine facilities. Additionally, this possibility facilitates logistics at the day of surgery.

An updated version of SPIO (SiennaXP^®^) is now tested in volumes of 1.5 and 1.0 ml without dilution.^15^ This might further reduce skin staining, and a smaller dose with peritumoral injection also might reduce artefacts in postoperative magnetic resonance imaging (MRI), because as a small volume around the lesion is easier to include in the surgical specimen.^16^,^17^ To investigate possible logistic benefits, a randomized trial is ongoing; either a magnetic clip is inserted for localisation of nonpalpable lesions^18^–^20^ at the same time as the SPIO or the SPIO is injected by the surgeon at the outpatient clinic and a guidewire is placed at the day of surgery. The clip and SPIO are inserted at any time within 4 weeks before surgery by the mammographist. Another implementation with promising preliminary results is from the ongoing SentiNot trial. The SPIO is injected at the time of surgery in patients with a preoperative diagnosis of ductal breast carcinoma in situ (DCIS). Then, the SN is removed at a second procedure, only if invasive cancer is detected on definitive histopathology.^21^ Finally, preliminary data depict that SPIO properties might pave the way towards the possibility for noninvasive axillary mapping.^22^

SPIO is a tracer with comparable results with the standard dual technique. Its benefits lie in the flexibility that it provides, which is an important property in the global setting where access to the isotope is difficult and in interesting clinical applications that are currently investigated. Refinement of the technique is expected to lead to optimal results and address issues with MRI follow-up compatibility, providing the possibility for a new standard in axillary mapping for breast cancer.
